# National Trends in Hospital Readmission Rates among Medicare Fee-for-Service Survivors of Mitral Valve Surgery, 1999–2010

**DOI:** 10.1371/journal.pone.0132470

**Published:** 2015-07-06

**Authors:** John A. Dodson, Yun Wang, Karthik Murugiah, Kumar Dharmarajan, Zack Cooper, Sabet Hashim, Sudhakar V. Nuti, Erica Spatz, Nihar Desai, Harlan M. Krumholz

**Affiliations:** 1 Leon H. Charney Division of Cardiology, Department of Medicine, New York University School of Medicine, New York, New York, United States of America; 2 Center for Outcomes Research and Evaluation, Yale New Haven Hospital, New Haven, Connecticut, United States of America; 3 Department of Biostatistics, Harvard School of Public Health, Boston, Massachusetts, United States of America; 4 Section of Cardiovascular Medicine, Yale University School of Medicine, New Haven, Connecticut, United States of America; 5 Department of Health Policy and Management, Yale School of Public Health, New Haven, Connecticut, United States of America; 6 Section of Cardiac Surgery, Department of Surgery, Yale University School of Medicine, New Haven, Connecticut, United States of America; 7 RWJ Clinical Scholars Program, Yale University School of Medicine, New Haven, Connecticut, United States of America; Harvard Medical School, UNITED STATES

## Abstract

**Background:**

Older patients who undergo mitral valve surgery (MVS) have high 1-year survival rates, but little is known about the experience of survivors. Our objective was to determine trends in 1-year hospital readmission rates and length of stay (LOS) in these individuals.

**Methods:**

We included 100% of Medicare Fee-for-Service patients ≥65 years of age who underwent MVS between 1999–2010 and survived to 1 year (N = 146,877). We used proportional hazards regression to analyze the post-MVS 1-year readmission rate in each year, mean hospital LOS (after index admission), and readmission rates by subgroups (age, sex, race).

**Results:**

The 1-year survival rate among patients undergoing MVS was 81.3%. Among survivors, 49.1% experienced a hospital readmission within 1 year. The post-MVS 1-year readmission rate declined from 1999–2010 (49.5% to 46.9%, P<0.01), and mean hospital LOS decreased from 6.2 to 5.3 (P<0.01). Readmission rates were highest in oldest patients, but declined in all age subgroups (65–74: 47.4% to 44.4%; 75–84: 51.4% to 49.2%, ≥85: 56.4% to 50.0%, all P<0.01). There were declines in women and men (women: 51.7% to 50.8%, P<0.01; men: 46.9% to 43.0%, P<0.01), and in whites and patients of other race, but not in blacks (whites: 49.0% to 46.2%, P<0.01; other: 55.0% to 48.9%, P<0.01; blacks: 58.1% to 59.0%, P = 0.18).

**Conclusions:**

Among older adults surviving MVS to 1 year, slightly fewer than half experience a hospital readmission. There has been a modest decline in both the readmission rate and LOS over time, with worse outcomes in women and blacks.

## Introduction

Mitral valve disease (most often regurgitation) is common in older adults [1]. In the subset of patients with severe symptomatic mitral regurgitation, definitive therapy involves repair or replacement of the mitral valve. Our group [2] and others [3] have shown a significant decline in U.S. mortality rates after mitral valve surgery (MVS) over time; for example, in Medicare beneficiaries ≥65 years undergoing isolated MVS over a 10-year period, risk-adjusted mortality declined by 40%, and by 2008 seven out of every eight patients undergoing MVS were alive at 1 year [2].

While this trend is encouraging, as the vast majority of MVS patients now survive, mortality statistics reveal little about the heterogeneity of experience among survivors. Survival is a successful outcome, but freedom from acute events that lead to hospitalizations is also important [4–6]. Do patients who survive after MVS tend to have repeated hospitalizations? How many days do they spend in the hospital? What are the causes of their hospitalizations? Data on these events in MVS survivors could be helpful in informing operative candidates about their risks after surgery and assist physicians and patients in making informed decisions and developing interventions to improve outcomes.

Accordingly, we sought to describe the overall hospital readmission rate and mean post-discharge length of stay (LOS) among U.S. older adults who survived MVS beyond 1 year, as well as trends in these outcomes over time, by analyzing data from 100% of Medicare fee-for-service (FFS) beneficiaries over a 12-year time period. The inclusion of all FFS beneficiaries allows for the establishment of a national benchmark for these outcomes, and also provides sufficient sample size to identify meaningful trends in potentially high-risk subgroups of age, sex, and race. To further characterize the experience among MVS survivors we analyzed discharge disposition, reasons for readmission, and outcomes by type of surgery.

## Patients and Methods

### Data Source and Coding

We used Medicare inpatient claims from the Centers for Medicare and Medicaid Services (CMS) to identify all Medicare Fee-for-Service patients discharged from an acute care hospital in the United States between January 1, 1999 and December 31, 2010, for a MVS procedure defined by the International Classification of Diseases, Ninth Revision, Clinical Modification (ICD-9-CM) codes of 35.12 (mitral vale repair), 35.23 (bioprosthetic mitral valve replacement), or 35.24 (mechanical mitral valve replacement). We further defined a subgroup of patients undergoing “isolated mitral valve surgery” by excluding patients with the following coexisting codes: CABG (36.10–36.17, 36.19), aortic valve repair/ replacement (35.11, 35.21, 35.22), tricuspid valve repair/replacement (35.14, 35.27, 35.28), endocarditis (421.0, 421.1, 421.9), or cardiogenic shock (785.51). If a patient had more than one MVS during an index year, the first one was selected. Patients were included in the final analytic sample if they underwent MVS and survived to 1 year following the index MVS hospitalization. We excluded those who were less than 65 years of age, or resided outside of the 50 US states, the District of Columbia, or Puerto Rico. We used 2011 Medicare claims data for patients who underwent MVS during 2010 to permit complete 1-year follow-up.

Institutional review board approval was obtained through the Yale University Human Investigation Committee, and the requirement for informed consent was waived based on the nature of the study. Beneficiary confidentiality was protected through a data use agreement with CMS. Patient information was anonymized but not de-identified prior to analysis. However, all analyses were conducted in a restricted office that meets CMS patient information and data security requirements.

### Patient Characteristics and Comorbidities

Patient demographic information includes age, sex, and race (white, black, other). Comorbidities were drawn from those used to profile hospitals by the CMS 30-day mortality measures for acute myocardial infarction [7] heart failure [8], and pneumonia [9]. They were identified from secondary diagnosis codes recorded at time of discharge from hospitalization for mitral valve surgery (which did not represent a potential complication) as well as primary or secondary diagnosis codes of all inpatient hospitalizations up to 1 year before hospitalization for surgery. Data from 1998 were used for patients hospitalized for MVS in 1999.

### Outcomes

The primary outcome was 1-year all-cause hospital readmission, defined as patients who had at least one rehospitalization within 1 year after surgery among patients who survived to 1 year. The "time zero" for a 1-year all-cause readmission was the date of the index MVS discharge. Secondary outcomes were the aggregate length of stay (LOS) (days) for all readmissions following index hospitalization for MVS, and total Medicare payments, within 1-year post-MVS. Post-MVS rehospitalization LOS represented the number of days spent in the hospital after the index MVS admission, and was calculated as the cumulative LOSs occurring after the index MVS hospitalization per MVS survivor. We report the mean LOS among those who were readmitted. The 1-year post MVS Medicare payments represented the inpatient Medicare expenditures (Medicare Part A) per MVS patient within the first year of MVS. We reported unadjusted dollar amounts and then adjusted using the annual Consumer Price Index inflation rate reported by the Bureau of Labor Statistics, United Stated Department of Labor (http://www.bls.gov/data/inflation_calculator.htm).

### Statistical Analysis

We examined patient demographic and clinical characteristics across years. The 1-year readmission rate was expressed as percentage; Medicare expenditures and length of stay as means (standard deviation [SD]). We used the Cochran-Armitage trend test to determine if changes over time in the outcomes were statistically significant. Data for each year of observation were included in this test; for purposes of simplicity, we report rates for first (1999) and last (2010) years in the text of the Results. We fitted a mixed model with a logit link function to estimate the changes in the 1-year rehospitalization rate after MVS over time, adjusting for patient demographics and comorbidities. We estimated the model with hospital-specific random intercepts to account for within-hospital and between-hospital sources of variation. We included an ordinal time variable, ranging from 0 to 10, corresponding to years 1999 (time = 0) to 2010 (time = 11), in the model. The odds ratios of this time variable represent the adjusted annual change in the 1-year readmission rate. We repeated the model for age and gender-race groups. All statistical testing was 2-sided, at a significance level of 0.05. Analyses were conducted using SAS version 9.3 64-bit (SAS Institute Inc, Cary, North Carolina).

## Results

### Patient characteristics

Of 180,568 patients undergoing MVS between 1999 and 2010, there were 146,877 (81.3%) who survived to 1 year. The 1-year survival rate increased over time (1999: 79.8%; 2010: 84.2%, P<0.001). Improved survival was seen across categories of age, sex, and race (1999–2010: 65–74: 82.9% to 87.2%; 75–84: 77.5% to 82.5%; ≥85: 67.7% to 75.4%; Male: 81.0% to 85.9%; Female: 78.8% to 82.6%; Black: 73.8% to 79.2%; White: 80.3% to 84.5%; Other: 73.0% to 84.6%;). Overall, patients who survived to 1 year were younger (74.8 vs. 76.3), more likely to be male (48.3% vs. 43.3%), white (92.2% vs. 90.1%), and had a lower prevalence of heart failure (26.7% vs. 38.9%) compared with those who did not survive to 1 year (all p values<0.001). We restricted the remainder of our analysis to 1-year MVS survivors.

The characteristics of 1-year MVS survivors over time are shown in Table 1. Between 1999 and 2010, the mean (SD) age of the survivors increased from 74.4 (5.2) to 75.0 (6.0) years, and the proportion of female patients decreased from 54.3% to 49.7%. There was a decrease in the prevalence of coronary artery disease (50.7% to 44.6%) while an increasing number of patients had hypertension (44.3% to 55.5%) and renal failure (2.6% to 10.5%), all p values <0.001. Isolated MVS represented 39.3% of all procedures over the study period. The mean LOS during index admission for surgery decreased from 11.2 days in 1999 to 10.8 days in 2010 (P<0.01).

**Table 1:** Characteristics of patients surviving 1 year after mitral valve surgery, 1999–2010.

Description	1999–2000	2001–2002	2003–2004	2005–2006	2007–2008	2009–2010
	N = 23,605	N = 25,276	N = 26,668	N = 25,819	N = 23,149	N = 22,360
**Demographics**						
Age, y, mean (SD)	74.5 (5.3)	74.6 (5.4)	74.8 (5.6)	74.8 (5.7)	75.0 (5.9)	75.0 (6.0)
Female (%)	54.5	53.7	51.7	50.2	49.7	50.3
White (%)	93.5	92.7	92.2	91.7	91.7	91.1
Black (%)	3.5	4.2	4.2	4.5	4.6	4.7
Other (%)	2.9	3.0	3.6	3.7	3.8	4.2
**Cardiovascular History (%)**						
Congestive heart failure	27.9	26.9	26.8	26.0	25.9	26.5
Prior myocardial infarction	4.9	4.9	4.9	4.3	4.6	4.1
Unstable angina	5.0	4.3	3.7	3.1	2.7	2.2
Coronary artery disease	51.4	52.6	52.8	50.5	49.6	47.1
Hypertension	45.6	50.8	52.1	51.9	54.0	55.0
Stroke	1.2	1.2	1.2	1.3	1.4	1.4
Cerebrovascular disease	3.3	3.2	3.2	2.8	3.0	2.7
Peripheral vascular disease	4.4	4.4	5.3	5.2	5.3	5.0
**Other Medical History (%)**						
Diabetes	16.8	17.9	18.5	18.9	18.8	17.8
Respiratory failure	3.2	3.2	3.2	4.0	5.3	5.6
Renal failure	2.7	3.2	4.2	6.1	9.0	10.0
COPD	18.8	20.1	20.7	21.6	18.3	14.7
Pneumonia	8.4	8.7	9.3	9.7	11.2	11.8
Cancer	4.4	4.4	4.7	4.5	4.8	4.5
Trauma	2.4	3.0	3.4	3.7	3.5	3.1
Liver disease	0.5	0.5	0.5	0.6	0.6	0.6
Depression	2.8	3.2	3.6	3.6	3.3	3.5
Other psychiatric disease	0.9	0.9	0.9	0.7	1.1	1.1
**Geriatric Conditions (%)**						
Malnutrition	1.7	1.8	2.2	2.6	4.0	5.0
Dementia	0.9	0.9	1.0	1.0	1.1	1.2
Functional disability	0.9	1.0	0.9	0.9	1.0	1.1

### 1-year readmission rate after MVS

**Fig 1:**
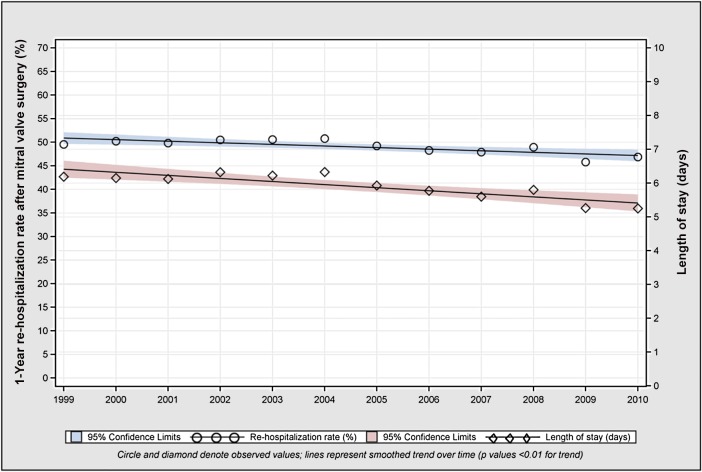
Overall trends in 1 year readmission rate and mean post-discharge length of stay in patients surviving to 1 year after mitral valve surgery, 1999–2010. The 1 year readmission rate declined from 49.5% (1999) to 46.9% (2010), and 1 year length of stay declined from 6.2 days (1999) to 5.3 days (2010).

Overall, 49.1% of patients surviving MVS to 1 year were readmitted at least once within the 1-year period. There was a modest decline in the overall observed 1-year readmission rate [95% CI] from 1999 to 2010 (49.5% [48.6–50.4] to 46.9% [45.9–47.8]) (Fig 1 and Table A in S1 File). In 1999, the top four readmission diagnoses were heart failure (17.9%), arrhythmia (10.8%), postoperative complication (6.6%), and pneumonia (3.2%). In 2010, heart failure (20.2%) and arrhythmia (12.2%) remained the most common readmission diagnoses, followed by sepsis (4.4%) and postoperative complication (4.0%). Among those who were readmitted within 1 year, median (IQR) number of days to first readmission was 39 (123) in 1999 and 38 (124) in 2010 (P = 0.57).

Over the aggregate study period, patients with mechanical MVR were the most likely to experience readmission (54.8%), followed by bioprosthetic MVR (51.8%) then MV repair (45.7%). Readmission rates over time stratified by valve subtype are shown in Table B in S1 File.

**Fig 2:**
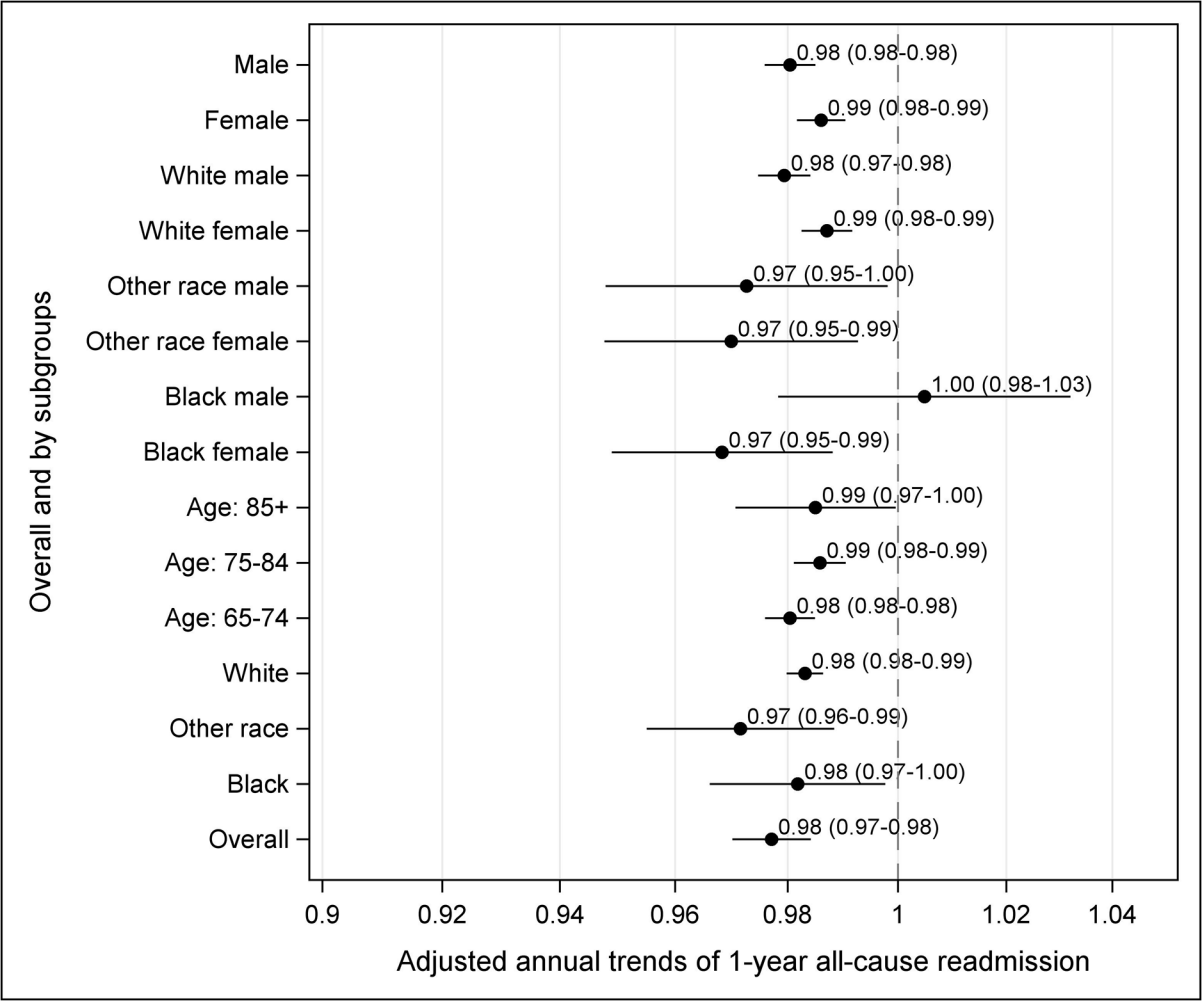
Risk-adjusted annual odds of readmission in patients surviving 1 year after mitral valve surgery, 1999–2010 (overall and by subgroups).

Among age subgroups, patients ≥85 years were the most likely to be readmitted within 1 year during all years of observation. Observed readmission rates [95% CI] declined in all ages between 1999 and 2010 (65–74: 47.4% [46.1–48.7] to 44.4% [43.0–45.7]; 75–84: 51.4% [50.1–52.8] to 49.2% [47.8–50.6]; ≥85: 56.4% [51.5–61.2] to 50.0% [41.2–53.8]) (Table A in S1 File). Women were more likely to experience 1-year readmission than men, although declines were seen in both groups (women: 51.7% [50.5–53.0] to 50.8% [49.5–52.1]; men: 46.9% [45.6–48.3] to 43.0% [41.7–44.3]). Among race subgroups, black patients had the highest 1-year readmission rates, and there was no decline in observed 1-year readmission over time (58.1% [53.1–62.9] to 59.0% [54.6–63.4]). Among patients of white and other races there were declines in 1-year readmission rates (white: 49.0% [48.1–50.0] to 46.2% [45.2–47.2]; other: 55.0% [49.4–60.4] to 48.9% [44.3–53.6]). The above findings did not change substantially after accounting for patient characteristics and within and between-hospital sources of variation, except for black patients, which after adjustment showed a statistically significant decline. Overall, the adjusted annual decline was 2.3% (95% CI 1.6 to 3.0). Fig 2 shows the adjusted annual decline in 1-year readmission by age-gender-race subgroup and overall.

In the isolated MVS cohort, 1-year readmission rates were generally lower in each year (Table A in S1 File). Similar to the full MVS cohort, there was a decline in 1-year readmission rates over time (45.9% [44.5–47.3] in 1999 to 42.8% [41.5–44.2] in 2010).

### Length of stay and discharge disposition after MVS

**Fig 3:**
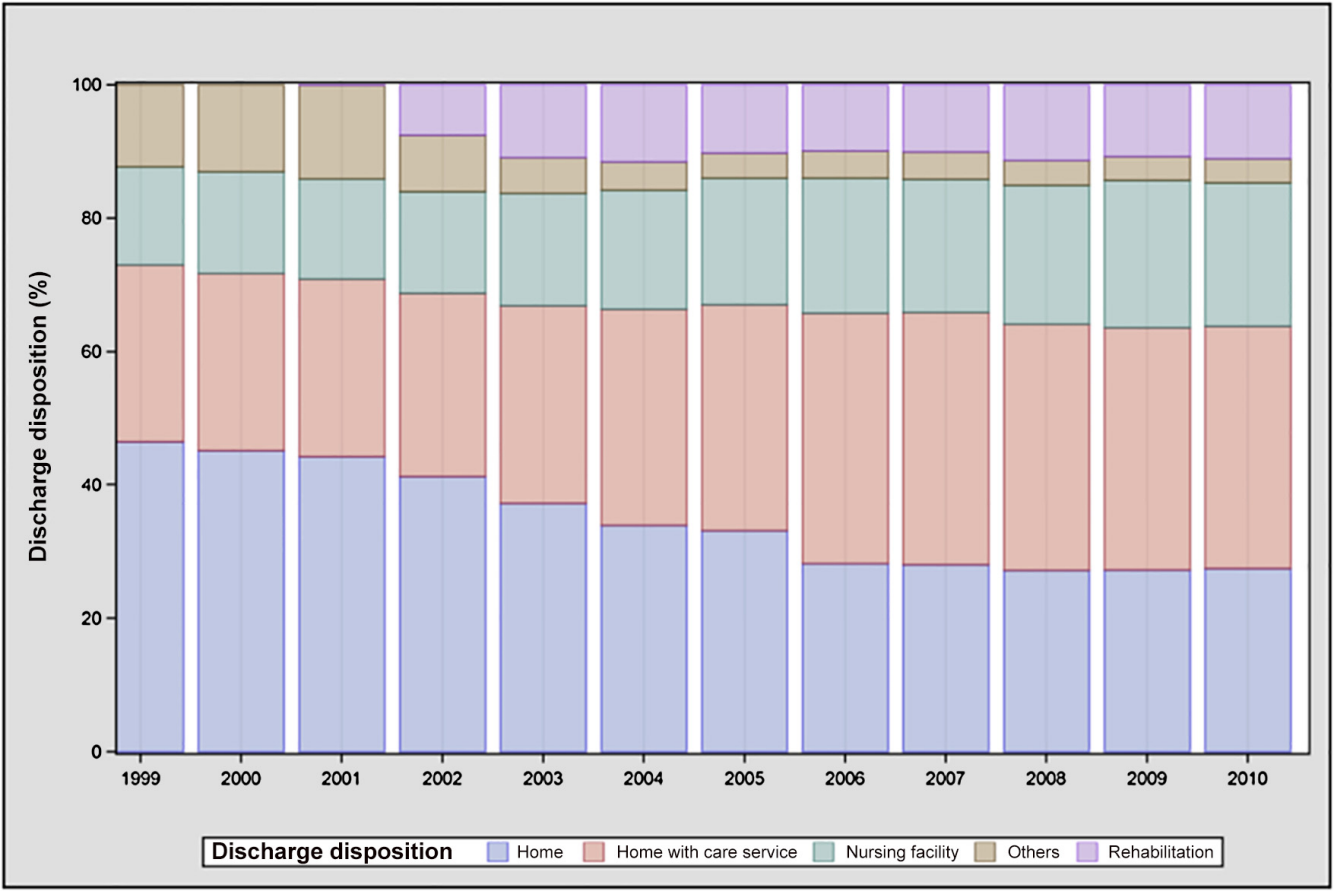
Discharge disposition (from index hospitalization) among patients surviving mitral valve surgery to 1 year, 1999–2010.

Mean hospital LOS [SD], excluding the index hospitalization, within the 1 year following MVS discharge decreased from 1999 to 2010 (6.2 [12.8] to 5.3 [11.7] days, P<0.001) (Table C in S1 File). LOS declined among all subgroups of age (65–74: 5.9 [13.3] to 5.0 [11.7] days; 75–84: 6.4 [12.0] to 5.4 [11.6] days; ≥85: 7.8 [14.1] to 6.2 [12.5] days, all P<0.001) and sex (women: 6.8 [13.2] to 5.9 [11.8] days; men: 5.5 [12.2] to 4.6 [11.5] days, all P<0.01). LOS did not decline significantly among black patients (9.9 [17.3] to 9.7 [18.0] days, p = 0.81), but declined among patients of white and other races (white: 6.0 [12.4] to 5.0 [11.1] days; other 7.7 [16.0] to 6.0 [13.7] days, all P<0.01). Between 1999 and 2010, the proportion of patients after MVS discharged directly to home without home care declined (46.5% to 27.5%); more patients were discharged to home with home care (26.5% to 36.3%); and an increasing number were discharged to skilled nursing or rehabilitation facilities (14.7% to 21.5%, all p<0.001 for trend, Fig 3). Over the aggregate study period, patients discharged to a home setting were less likely to experience readmission within 1 year of discharge compared with patients discharged elsewhere (42.0% vs. 58.0%, P<0.001). The percentage of patients who spent more than 30 days in the hospital after discharge was low throughout the study period and declined over time (1999: 4.3% [4.0–4.7]; 2010: 3.4%[3.1–3.8]).

In the isolated MVS cohort, mean hospital LOS [SD] declined from 5.3 (11.5) days in 1999 to 4.4 (9.5) days in 2010 (P<0.001). LOS declined significantly in all subgroups except patients of black and other race (black: 9.6 [16.9] to 8.0 [16.5] days, p = 0.36; other: 5.3 [10.6] to 6.5 [12.9] days, p = 0.36). Over time isolated MVS patients were less frequently discharged directly to home (1999: 56.1%; 2010: 41.5%), and more often discharged to home with home care (1999: 13.8%; 2010: 18.7%) or skilled nursing or rehabilitation (1999: 8.6%; 2010: 16.0%) (all p values <0.01).

### Trends in CMS payments

Based on the number of patients who were hospitalized in the year following MVS in 2010 (5,151/10,983), we calculated the increase in total cost compared with the equivalent number of patients hospitalized in 1999. The average unadjusted CMS payment per patient for all readmissions in the 1 year following MVS (total surgeries) increased from $40,730 in 1999 to $56,922 in 2010, a net change of 39.8%. After adjusting for inflation (2010 dollars), the rise is cost was more modest: $53,310 in 1999 and $56,922 in 2010 (relative change of 6.8%). For reference, the unadjusted growth in overall health spending over this period was approximately 50%.

## Discussion

Using a 100% sample of Medicare fee-for-service beneficiaries we found that slightly fewer than half of older adults who underwent MVS and survived to 1 year were readmitted to the hospital. Over time there was a modest decline in the readmission rate, despite findings of an increasing age and comorbidity burden among the population. In addition, over the same period we found that fewer days on average (following the surgical admission) were spent in the hospital; by 2010 the mean number of days had declined to 5.3 (compared with 6.2 in 1999). Furthermore, there was a decline in the proportion of patients with extended hospital stays; in 2010, fewer than 1 in 25 patients spent more than 30 days in the hospital. Reasons for readmission were heterogeneous; in both 1999 and 2010 the top two diagnostic codes were heart failure and arrhythmia, although together they only accounted for 28.7% and 32.4% of the total readmissions in each year, respectively.

Several factors may explain the modest decline in readmissions. We found that over time, fewer patients were discharged directly to home without home care, and more were discharged either home with home care (relative increase 39%) or to skilled nursing or rehabilitation facilities (relative increase 86%). Similar trends have previously been described in Medicare patients undergoing CABG [10] and those hospitalized for heart failure [11]. It is possible that for patients undergoing MVS, the increased post-discharge attention provided in these settings facilitated interventions aimed at addressing preventable or unnecessary readmissions. Further study would be necessary to support this relationship; for other conditions such as heart failure and acute myocardial infarction, data on the potential for skilled nursing facility referral to reduce readmissions have been mixed [12,13]. Another factor in the reduced readmission rate among 1-year survivors may be the increasing focus of cardiac surgical teams in reducing sternal wound infections [14] and pneumonia [15] among patients undergoing open heart surgery, which may have reduced readmissions related to hospital-acquired infections over time. Technological advances in operative techniques, particularly an increasing adoption of mitral valve repair over time (obviating the need for long-term oral anticoagulation), may also have contributed. It is also possible that patients referred for surgery were healthier; while we found that age and comorbidities increased, which parallels findings of others [3], it has also been reported that preoperative New York Heart Association (NYHA) functional class among MVS candidates has improved over time [16], suggesting a trend towards earlier intervention.

There were important differences among subgroups that persisted over the study period. Over all years, black patients had the highest rates of readmission. This parallels findings of higher readmission rates for black patients after other cardiac procedures including CABG [17] and percutaneous coronary intervention [18]. Given the administrative nature of our data we are limited in determining causal factors, though prior studies among patients undergoing MVS have reported black patients having higher rates of postoperative prolonged mechanical ventilation and renal failure [19] which may contribute to an increased vulnerability to readmission after discharge. Other system-level factors may also have played a role such as site of hospital care [20] or poor access to early outpatient follow-up care after discharge [21], as reported for other conditions. We also found that the oldest patients (≥85 years) had a higher rate of 1-year readmissions than younger age groups. While this may seem intuitive due to the general prevalence of comorbidities and frailty among this population, other studies have found no relationship between age and readmission after hospitalizations for heart failure [22] or general surgical procedures [23]. Finally, patients who underwent mechanical MVR were more likely to experience readmission than patients with MV repair or bioprosthetic MVR, which may be related to bleeding complications associated with mandatory anticoagulant therapy, or alternately due to other patient characteristics such as advanced heart disease (both technically prohibiting MV repair and leading to increased vulnerability for hospital readmission). Further studies with more granular data would be required to confirm such a relationship.

Our findings must be interpreted in the context of our study design. First, our analysis was limited to Medicare FFS beneficiaries and we therefore do not have data on patients enrolled in Medicare managed care programs, where enrollment has increased over time [24]. Similarly, we do not have data on patients younger than age 65 undergoing MVS. Second, our study sample was restricted to MVS survivors, and since mortality rates have decreased over time, the nature of patients surviving MVS (and at-risk for readmission) may have changed. However, the absolute decrease in mortality over time was relatively small. Third, since we relied on administrative data it is possible that coding of comorbidities changed over time (e.g. up-coding or down-coding), which could have affected our ability to adjust for potential confounders. With our administrative dataset we were also unable to account for certain selection attributes that may have influenced the choice of patients for surgery, and we could not adjust for preoperative data such as ejection fraction and symptom severity which may have influenced outcomes. Finally, beyond categories of age, sex, and race, our study was not designed to determine risk factors for post-MVS readmission. Readmission events are likely to represent a complex interaction of patient-level factors (including specific vulnerabilities to acute medical illness) and system-level characteristics (such as the quality of in-hospital and post-hospital care). Elucidating the determinants of post-hospital risk remains an important direction for future investigation.

In conclusion, we found that slightly fewer than half of MVS 1-year survivors experienced a hospital readmission. While there was a modest decline in this rate over time, there is still considerable room for interventions that may further reduce preventable events. Our finding of considerable variation among subgroups requires further study and suggests targeted areas for quality improvement.

## Supporting Information

S1 File This file contains Tables A-C. Trends in readmission rate among mitral valve surgery survivors at 1 year, 1999–2010 (Table A). Trends in 1-year readmission by surgical subtype among 1 year MVS survivors, 1999–2010 (Table B). Trends in mean total length of stay (within 1 year of discharge) among mitral valve surgery survivors at one year, 1999–2010 (Table C).
(PDF)
