# Supplementary material for: National Trends in Hospital Readmission Rates among Medicare Fee-for-Service Survivors of Mitral Valve Surgery, 1999–2010
Source: PLoS One. 2015 Jul 6;10(7):e0132470. doi: 10.1371/journal.pone.0132470 (PMC4493110; doi:10.1371/journal.pone.0132470)
Supplement: S1 File — Trends in readmission rate among mitral valve surgery survivors at 1 year, 1999–2010 (Table A). Trends in 1-year readmission by surgical subtype among 1 year MVS survivors, 1999–2010 (Table B). Trends in mean total length of stay (within 1 year of discharge) among mitral valve surgery survivors at one year, 1999–2010 (Table C). (PDF) [file pone.0132470.s001.pdf]

SUPPORTING INFORMATION

S1 File. Trends in readmission rate among mitral valve surgery survivors at 1 year, 1999-2010 (Table A). Trends in 1-year readmission by surgical subtype among 1 year MVS survivors, 1999-2010 (Table B). Trends in mean total length of stay (within 1 year of discharge) among mitral valve surgery survivors at one year, 1999-2010 (Table C).

Table A. Trends in readmission rate among mitral valve surgery survivors at 1 year, 1999-2010

**Description**	1999	2000	2001	2002	2003	2004	2005	2006	2007	2008	2009	2010
**Full MVS Cohort**	N = 11,492	N = 12,113	N = 12,166	N = 13,110	N = 13,272	N = 13,396	N = 13,411	N = 12,408	N = 11,744	N = 11,405	N = 11,377	N = 10,983
At least one re-hospitalization, %, (95% CI)	49.5 (48.6-50.4)	50.2 (49.3-51.1)	49.8 (48.9-50.7)	50.5 (49.6-51.4)	50.6 (49.7-51.4)	50.8 (49.9-51.6)	49.2 (48.3-50.0)	48.3 (47.4-49.2)	47.9 (47.0-48.8)	49.0 (48.0-49.9)	45.8 (44.9-46.7)	46.9 (45.9-47.8)
By age												
65-74	47.4 (46.1-48.7)	48.0 (46.7-49.2)	47.5 (46.3-48.8)	48.8 (47.6-50.0)	48.8 (47.6-50.1)	48.2 (47.0-49.4)	46.9 (45.7-48.1)	45.5 (44.3-46.8)	45.8 (44.5-47.1)	46.4 (45.1-47.8)	42.6 (41.3-44.0)	44.4 (43.0-45.7)
75-84	51.4 (50.1-52.8)	52.3 (50.9-53.6)	51.9 (50.6-53.2)	52.1 (50.8-53.3)	51.6 (50.4-52.9)	52.7 (51.4-53.9)	51.2 (49.9-52.4)	50.6 (49.3-52.0)	49.7 (48.3-51.0)	50.7 (49.4-52.1)	48.0 (46.6-49.3)	49.2 (47.8-50.6)
≥85	56.4 (51.5-61.2)	55.5 (50.9-60.0)	55.1 (50.5-59.7)	55.1 (50.5-59.6)	58.8 (54.7-62.9)	58.5 (54.4-62.5)	54.2 (50.2-58.2)	53.7 (49.7-57.6)	51.5 (47.5-55.6)	56.4 (52.5-60.3)	55.0 (51.2-58.7)	50.0 941.2-53.8)
By sex												
Female	51.7 (50.5-53.0)	53.0 (51.8-54.2)	52.1 (50.9-53.4)	52.7 (51.5-53.9)	52.7 (51.5-53.8)	53.2 (52.0-54.4)	51.6 (50.4-52.8)	51.4 (50.1-52.6)	50.6 (49.3-51.9)	51.9 (50.6-53.2)	49.1 (47.8-50.4)	50.8 (49.5-52.1)
Male	46.9 (45.6-48.3)	46.8 (45.5-48.2)	47.1 (45.7-48.4)	47.9 (46.7-49.2)	48.3 (47.0-49.5)	48.2 (47.0-49.4)	46.8 (45.6-48.0)	45.1 (43.9-46.4)	45.2 (43.9-46.5)	46.1 (44.8-47.4)	42.4 (41.1-43.7)	43.0 (41.7-44.3)

By race												
Black	58.1 (53.1-62.9)	62.9 (58.2-67.5)	60.0 (55.5-64.3)	62.0 (57.9-66.0)	60.5 (56.3-64.6)	58.4 (54.3-62.5)	57.0 (53.0-61.0)	59.9 (55.7-64.0)	58.9 (54.5-63.1)	57.7 (53.4-62.0)	59.4 (55.1-63.5)	59.0 (54.6-63.4)
White	49.0 (48.1-50.0)	49.6 (48.7-50.5)	49.3 (48.4-50.2)	49.8 (48.9-50.7)	50.1 (49.2-51.0)	50.2 (49.3-51.1)	48.8 (48.0-49.7)	47.6 (46.7-48.5)	47.4 (46.5-48.4)	48.4 (47.5-49.4)	44.9 (44.0-45.9)	46.2 (45.2-47.2)
Other	55.0 (49.4-60.4)	54.2 (48.9-59.4)	51.7 (46.1-57.2)	54.1 (49.3-58.8)	51.4 (46.8-55.9)	54.8 (50.3-59.3)	48.5 (44.1-52.9)	50.3 (45.6-55.0)	46.9 (42.1-51.8)	50.3 (45.6-55.0)	49.3 (44.7-53.9)	48.9 (44.3-53.6)
**Isolated MVS Cohort**	N = 4946	N = 5204	N = 5154	N = 5390	N = 5377	N = 5367	N = 5287	N = 4857	N = 4790	N = 4684	N = 5011	N = 5109
Readmission rate (total), %	45.9 (44.5-47.3)	47.8 (46.5-49.2)	47.1 (45.7-48.5)	47.2 (45.8-48.5)	46.8 (45.5-48.1)	47.9 (46.6-49.3)	44.2 (42.9-45.6)	44.6 (43.2-46.0)	43.6 (42.2-45.0)	45.0 (43.6-46.4)	41.8 (40.5-43.2)	42.8 (41.5-44.2)
By age												
65-74	44.2 (42.3-46.0)	45.3 (43.5-47.2)	44.1 (42.2-45.9)	45.3 (43.5-47.2)	45.2 (43.4-47.1)	44.6 (42.7-46.5)	41.5 (39.6-43.3)	41.2 (39.3-43.1)	41.0 (39.1-43.0)	42.2 (40.3-44.2)	38.2 (36.3-40.1)	39.3 (37.5-41.2)
75-84	47.5 (45.3-49.7)	50.6 (48.5-52.7)	50.3 (48.2-52.4)	48.9 (46.8-50.9)	47.8 (45.7-49.8)	51.0 (49.0-53.0)	46.6 (44.5-48.7)	48.1 (45.9-50.3)	46.2 (44.1-48.4)	47.5 (45.3-49.7)	44.8 (42.7-46.9)	46.4 (44.3-48.5)
≥85	57.5 (49.3-65.5)	55.2 (47.5-62.7)	55.8 (47.8-63.5)	55.1 (47.2-62.8)	57.1 (50.1-63.8)	56.1 (49.3-62.8)	55.2 (48.4-61.9)	53.4 (46.3-60.4)	48.6 (41.1-56.1)	52.0 (45.6-58.3)	53.9 (47.7-60.0)	49.2 (43.1-55.4)
By sex												

Female	48.0 (46.1-49.8)	50.2 (48.4-51.9)	48.9 (47.1-50.6)	49.5 (47.8-51.2)	48.6 (46.8-50.3)	50.1 (48.4-51.9)	46.1 (44.3-47.9)	47.5 (45.6-49.4)	46.4 (44.5-48.3)	47.5 (45.5-49.4)	44.7 (42.9-46.6)	47.6 (45.7-49.5)
Male	42.8 (40.6-45.0)	44.1 (41.9-46.3)	44.5 (42.3-46.6)	43.6 (41.5-45.8)	44.2 (42.1-46.3)	45.0 (42.9-47.0)	41.9 (39.9-43.9)	40.6 (38.5-42.8)	40.0 (37.9-42.2)	41.9 (39.8-44.1)	38.2 (36.2-40.3)	37.2 (35.3-39.2)
By race												
Black	55.2 (47.5-62.8)	60.2 (53.2-66.9)	59.0 (52.1-65.8)	53.2 (46.6-59.8)	60.1 (53.3-66.6)	59.8 (53.3-65.9)	51.5 (44.8-58.2)	53.3 (46.3-60.1)	53.7 (46.8-60.6)	53.7 (47.0-60.4)	54.5 (47.5-61.4)	54.8 (48.0-61.6)
White	45.5 (44.0-46.9)	47.1 (45.6-48.5)	46.5 (45.1-48.0)	46.7 (45.3-48.1)	46.2 (44.8-47.6)	47.1 (45.7-48.6)	44.1 (42.7-45.6)	44.1 (42.6-45.6)	43.4 (41.7-44.6)	44.5 (43.0-46.0)	40.8 (39.4-42.3)	42.1 (40.7-43.5)
Other	48.1 (40.2-56.1)	56.8 (48.4-65.0)	47.9 (39.4-56.5)	51.5 (44.3-58.6)	46.1 (38.7-53.7)	53.0 (45.8-60.0)	37.8 (30.8-45.2)	46.6 (39.0-54.3)	41.9 (34.6-49.5)	46.9 (39.4-54.5)	51.1 (44.4-57.8)	47.0 (40.0-54.2)

Table B. Trends in 1-year readmission by surgical subtype among 1 year MVS survivors, 1999-2010

	1999	2000	2001	2002	2003	2004	2005	2006	2007	2008	2009	201[illegible]
Bioprosthesis	51.4%	52.9%	52.4%	53.3%	52.4%	52.1%	51.3%	51.1%	50.7%	53.1%	50.8%	50.5[illegible]
MV repair	46.5%	45.7%	47.2%	47.9%	48.7%	48.8%	46.4%	45.6%	44.7%	45.0%	40.7%	42.3[illegible]
Mechancal MVR	54.0%	54.7%	50.8%	53.0%	56.4%	56.4%	58.5%	52.0%	56.9%	57.4%	58.2%	53.6[illegible]

Table C. Trends in mean total length of stay (within 1 year of discharge) among mitral valve surgery survivors at one year, 1999-2010

**Description**	1999	2000	2001	2002	2003	2004	2005	2006	2007	2008	2009	2010
**Full MVS Cohort**	N = 11,492	N = 12,113	N = 12,166	N = 13,110	N = 13,272	N = 13,396	N = 13,411	N = 12,408	N = 11,744	N = 11,405	N = 11,377	N = 10,983
Mean days in hospital (±SD)	6.2 ± 12.8	6.2 ± 12.4	6.1 ± 12.9	6.3 ± 12.7	6.2 ± 12.1	6.3 ± 12.8	5.9 ± 12.0	5.8 ± 11.8	5.6 ± 11.8	5.8 ± 12.1	5.3 ± 11.3	5.3 ± 11.7
By age												
65-74	5.9 ± 13.3	5.8 ± 12.2	6.0 ± 13.5	6.0 ± 12.3	6.0 ± 12.3	6.1 ± 13.3	5.7 ± 12.2	5.4 ± 11.6	5.3 ± 11.8	5.5 ± 12.1	5.1 ± 11.8	5.0 ± 11.7
75-84	6.4 ± 12.0	6.5 ± 12.6	6.2 ± 11.9	6.7 ± 13.2	6.3 ± 12.0	6.5 ± 12.3	6.2 ± 11.9	6.0 ± 11.8	5.9 ± 11.7	6.0 ± 12.0	5.3 ± 10.8	5.4 ± 11.6
≥85	7.8 ± 14.1	6.8 ± 13.0	7.3 ± 16.1	6.7 ± 11.9	7.3 ± 11.8	7.0 ± 11.6	6.0 ± 9.3	7.1 ± 12.9	6.0 ± 11.6	7.0 ± 12.3	6.2 ± 11.2	6.2 ± 12.5
By sex												
Female	6.8 ± 13.2	6.7 ± 12.6	6.7 ± 13.5	7.0 ± 13.7	6.8 ± 12.5	7.1 ± 13.3	6.5 ± 12.4	6.5 ± 12.5	6.4 ± 12.5	6.8 ± 13.5	5.8 ± 11.7	5.9 ± 11.8
Male	5.5 ± 12.2	5.5 ± 12.2	5.5 ± 12.1	5.6 ± 11.4	5.6 ± 11.7	5.6 ± 12.1	5.3 ± 11.4	5.0 ± 11.0	4.8 ± 10.8	4.8 ± 10.3	4.8 ± 10.9	4.6 ± 11.5
By race												
Black	9.9 ± 17.3	9.5 ± 14.9	9.8 ± 16.4	11.2 ± 19.3	10.1 ± 17.1	9.4 ± 18.5	11.0 ± 19.8	10.4 ± 18.3	9.9 ± 17.4	10.2 ± 18.7	10.0 ± 16.8	9.7 ± 18.0
White	6.0 ± 12.4	6.0 ± 12.2	5.9 ± 12.6	6.0 ± 11.8	6.0 ± 11.8	6.1 ± 12.2	5.6 ± 11.2	5.5 ± 11.3	5.4 ± 11.4	5.5 ± 11.4	4.9 ± 10.6	5.0 ± 11.1
Other	7.7 ± 16.0	7.1 ± 15.6	7.7 ± 16.5)	9.2 ± 21.0	6.7 ± 12.3	8.1 ± 17.6	7.6 ± 15.3	6.3 ± 11.8	5.4 ± 10.9	7.42 ± 15.0	7.9 ± 17.0	6.0 ± 13.7

**Isolated MVS Cohort**	N = 4946	N = 5204	N = 5154	N = 5390	N = 5377	N = 5367	N = 5287	N = 4857	N = 4790	N = 4684	N = 5011	N = 5109
Mean days in hospital (±SD)	5.3 ± 11.5	5.7 ± 12.1	5.6 ± 11.7	5.6 ± 11.5	5.5 ± 11.1	5.5 ± 11.3	4.8 ± 10.9	4.8 ± 10.7	4.8 ± 10.8	4.9 ± 10.5	4.2 ± 9.3	4.4 ± 9.5
By age												
65-74	5.1 ± 12.0	5.4 ± 12.3	5.3 ± 12.2	5.2 ± 10.7	5.1 ± 10.7	5.2 ± 11.5	4.4 ± 10.7	4.2 ± 10.0	4.5 ± 10.9	4.3 ± 10.1	3.9 ± 9.6	4.0 ± 9.5
75-84	5.4 ± 10.7	6.0 ± 11.7	5.7 ± 10.4	6.0 ± 12.4	5.7 ± 11.3	5.9 ± 11.1	5.2 ± 11.4	5.3 ± 10.7	5.0 ± 10.4	5.2 ± 10.5	4.5 ± 8.7	4.8 ± 9.7
≥85	7.5 ± 11.6	6.3 ± 13.6	8.4 ± 17.4	6.9 ± 12.5	7.7 ± 12.9	5.7 ± 11.1	5.9 ± 9.5	7.9 ± 16.8	5.9 ± 14.7	7.2 ± 12.9	5.7 ± 9.5	5.2 ± 8.6
By sex												
Female	5.8 ± 11.9	6.0 ± 11.6	6.0 ± 12.6	6.1 ± 11.6	6.0 ± 11.4	6.0 ± 11.3	5.1 ± 11.0	5.6 ± 11.6	5.4 ± 11.4	5.6 ± 11.3	4.6 ± 9.5	5.2 ± 10.4
Male	4.6 ± 10.8	5.1 ± 12.8	4.9 ± 10.2	4.9 ± 11.4	4.8 ± 10.6	4.9 ± 11.2	4.4 ± 10.9	3.8 ± 9.1	3.9 ± 10.1	4.0 ± 9.4	3.7 ± 9.0	3.4 ± 8.3
By race												
Black	9.6 ± 16.9	10.1 ± 17.3	8.8 ± 16.1	10.3 ± 21.2	9.8 ± 15.9	8.2 ± 14.3	8.9 ± 16.8	9.0 ± 17.6	9.0 ± 15.4	9.0 ± 17.3	9.2 ± 15.9	8.0 ± 16.5
White	5.2 ± 11.3	5.4 ± 11.7	5.4 ± 11.5	5.3 ± 10.6	5.3 ± 10.8	5.3 ± 11.1	4.6 ± 10.4	4.6 ± 10.2	4.6 ± 10.6	4.6 ± 9.6	3.9 ± 8.6	4.1 ± 8.8
Other	5.3 ± 10.6	7.7 ± 12.8	5.5 ± 10.4	7.9 ± 16.4	5.0 ± 9.4	6.3 ± 12.7	4.9 ± 13.5	5.2 ± 10.9	4.5 ± 9.1	7.3 ± 15.9	6.6 ± 11.8	6.5 ± 12.9
